# Assessing the difficulty of forceps delivery using a transperineal ultrasonographic station: a prospective cohort study

**DOI:** 10.1007/s10396-025-01536-1

**Published:** 2025-04-09

**Authors:** Hiroko Takita, Ryu Matsuoka, Bunbu Sekiya, Yuki Mukai, Takeshi Nakamura, Mayumi Kaneko, Akihiko Sekizawa

**Affiliations:** Department of Obstetrics and Gynecology, Showa University School of Medicine, 1-5-8 Hatanodai, Shinagawa-ku, Tokyo, 142-8666 Japan

**Keywords:** Transperineal ultrasonography, Angle of progression, Digital vaginal examination, Forceps delivery, Fetal complications, Labour progression, Pelvic radiographs

## Abstract

**Purpose:**

To investigate the clinical utility of a novel transperineal ultrasonographic (TUS) parameter, the TUS station, for assessing labour progression and predicting forceps delivery difficulty.

**Methods:**

In this prospective cohort study, 384 TUS images from 144 participants who delivered vaginally between January 2019 and December 2021 were assessed for labour progression in a single tertiary perinatal center in Japan. Singleton cephalic pregnancies ≥ 37 weeks were included. The digital vaginal examination (DVE) findings, TUS station, and angle of progression (AoP), an ultrasound parameter commonly used for assessing labour progression, were obtained by individual obstetricians who were blinded to the results in all cases. The TUS station and DVE findings and AoP and DVE findings of the participants were compared. A second cohort requiring forceps delivery was analyzed to explore the relationship between TUS station and delivery difficulty.

**Results:**

In the quantitative assessment of fetal head descent, the TUS station correlated with DVE findings. During the resting phase of labour, the TUS cut-off value for an easy forceps delivery was 2 cm, with a sensitivity of 85%; the maximum AUC value was 0.9 (95% confidence interval [CI]: 0.73–0.96). At the time of labour, the TUS cut-off value for an easy forceps delivery was 2.9 cm, with a sensitivity of 85%; the maximum AUC value was 0.8 (95% CI 0.65–0.96).

**Conclusion:**

The TUS station may serve as a valuable and objective parameter for consideration in decisions regarding forceps delivery.

## Introduction

Labour progression is traditionally evaluated using digital vaginal examination (DVE), which uses DeLee’s definition to evaluate the degree of infant head descent [1, 2]. Subsequently, the American College of Obstetricians and Gynecologists (ACOG) station concept was developed based on DeLee’s station concept, with the ischial spines serving as the reference for station ± 0. However, DVE based on DeLee’s station relies on the operator’s experience and lacks objectivity [3–6].

An accurate assessment of labour progression is particularly crucial before instrumental deliveries. Forceps delivery requires more forceful traction than vacuum delivery and is more prone to maternal and fetal complications [7, 8]. The decision to perform forceps delivery is based on DVE findings. The Royal College of Obstetricians and Gynaecologists (RCOG) and ACOG recommend evaluation of the fetal head station, defined as the fetal head’s level in the birth canal relative to the maternal ischial spine plane [9], before forceps delivery. The plane passing through the ischial spine and posterior pubic bone is considered as station ± 0. The distance of head descent is evaluated as higher or lower head stations [9].

Recently, transperineal ultrasonography (TUS) has shown promise for the objective evaluation of labour progression. The International Society of Ultrasound in Obstetrics and Gynecology (ISUOG) has published practice guidelines for intrapartum ultrasonography [9]. The angle of progression (AoP), head-perineum distance, head direction (HD) for the pubic symphysis, and midline angle are typical TUS parameters assessed during the second stage of labour [9]. The AoP is widely used to assess the descent of a fetal head during delivery.

Using pelvic computed tomography (CT) three-dimensional images, Tutschek et al. determined that an AoP of 116° on TUS was equivalent to station ± 0 on DVE. They used vertical distance as the fetal head station from the lower margin of the pubic symphysis to the lowest bony part of the presenting head. We reported that the station ± 0 position is located at an angle of 119° from the long axis of the pubis, based on pelvic measurement using X-ray images of 501 Japanese pregnant women [10]. The station ± 0 defined by Tutschek is determined as the plane of Hodge, parallelly shifted about 30 mm from the inferior margin of the pubis, which results in a correlation with maternal body habitus. However, by defining the station ± 0 position based on the angle with the long axis of the pubis, our method eliminates this correlation with maternal body habitus. This finding indicates that station ± 0 exists on a plane at 119° regardless of maternal height. Furthermore, the birth canal is not a straight path but rather curved. Even Tutschek acknowledged that defining fetal descent using parallel planes creates a discrepancy from the physiological descent of the fetal head along the curved birth canal [11]. In contrast, our method directly measures the distance between station ± 0 and the leading part of the fetal head. This approach allows for an assessment along the birth canal even when the fetal head begins engagement in the third plane. Therefore, our measurement of fetal head descent using transperineal ultrasound provides a more physiologically accurate and objective assessment of fetal head descent. The calculated angle of 119° is derived from a large sample of Japanese pregnant women [10]. The TUS head station may be a more comprehensive fetal station parameter than the AoP.

This study aimed to demonstrate the usefulness of TUS station for fetal head station assessment and its predictive ability for the difficulty of forceps delivery.

## Material and methods

### Study design

This study comprised 384 TUS images from 144 participants who delivered vaginally between January 2019 and December 2021 in a single tertiary perinatal center in Japan. We included cases of singleton cephalic pregnancies of a gestational age (GA) ≥ 37 weeks.

In addition, we conducted a prospective analysis of another cohort of 117 pregnant women who required instrumental deliveries using Naegele forceps (Atom Medical Corporation, Tokyo, Japan) between March 2019 and December 2021. The 117 cases in this study did not include cases of abnormal internal rotation. The indications for forceps delivery were non-reassuring fetal status, prolonged labour, and delivery arrest. When forceps delivery was performed, the degree of head lowering was evaluated using DVE in all cases, and TUS was performed by a different physician from the forceps operator, and images were taken to assist in the evaluation. Our policy is to deliver forceps deliveries in a single procedure. Cases in which delivery could not be achieved by forceps delivery and a cesarean section was performed, or cases in which forceps delivery required two or more traction attempts, were defined as failed forceps delivery cases and classified as difficult forceps delivery (“difficult forceps delivery” cohort), whereas those requiring a single traction were classified as easy forceps delivery (“easy forceps delivery” cohort). Cases with missing ultrasound data were excluded. The final decision for forceps delivery was based on vaginal examination findings, while transperineal ultrasound findings were limited to observation and did not influence the decision-making process based on threshold values.

### TUS station ± 0

**Fig. 1:**
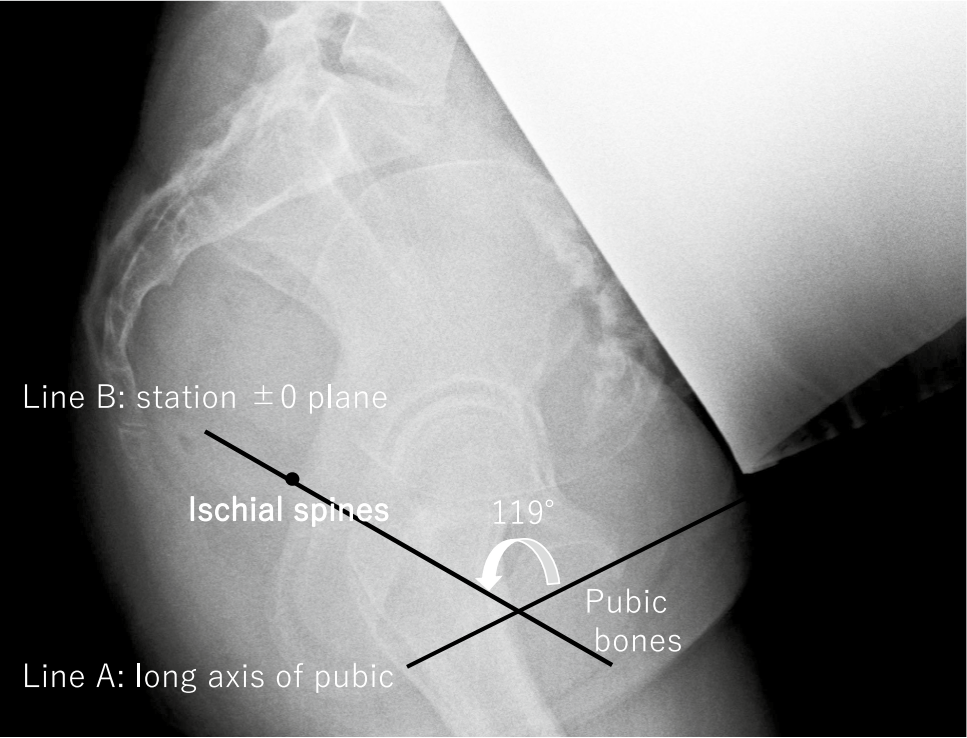
Identification of station ± 0 in pelvic radiography using the Guzman method. On Guzman X-ray picture, the line from the ischial spine to the lower margin of the pubic bone (line B) represents station ± 0. Line A is the long axis of the pubic symphysis. Line B passing through the ischial spine and the lower margin of the pubic bone is defined as “station ± 0”. The angle between lines A and B was measured. We detected the 119° line from line A as corresponding to station ± 0 as determined using digital vaginal examination (DVE) in Japanese women

**Fig. 2:**
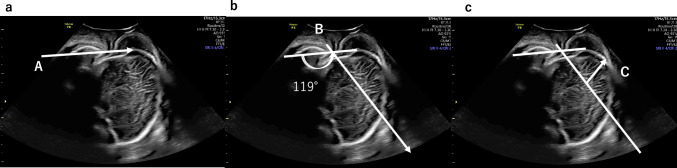
Procedure of transperineal ultrasound station measurement. The procedure for transperineal ultrasound station measurement was as follows: 1. The long axis of the pubic symphysis and the vertex of the fetal head are captured on the same screen. Line A is drawn to identify the long axis of the pubic symphysis. 2. Line B is drawn from the lower end of the pubic bone to create an angle of 119° with line A. Line B shows TUS station ± 0. 3. The distance is measured (arrow C) from the intersection of the TUS ± 0 line and the midline of the fetal head to the most advanced part of the fetal head along the direction of the head. The length of arrow C shows the TUS station

In our previous study on TUS station, we performed radiographic pelvic measurements using the Guthmann method in 501 Japanese patients with singleton cephalic pregnancies between 35 and 39 weeks of gestation [10]. Using pelvic radiographs, the pubic bone and ischial spine were identified (Fig. 1). The angle between the long axis of the pubic symphysis and the line connecting the lower margin of the pubic bone and ischial spine was defined as station ± 0. We detected 119° as TUS station ± 0, which corresponded to station ± 0 of DVE assessment in Japanese women. The method for visualizing TUS station ± 0 on transperineal ultrasound images is shown in Fig. 2. Similar to the AoP measurement, the pubis is displayed horizontally in the upper left of the screen in a pelvic midsagittal plane. Line A is drawn along the long axis of the pubis, and Line B is drawn from the inferior margin of the pubis at an internal angle of 119°. This Line B represents station ± 0 on transperineal ultrasound images. A perpendicular Line C is then drawn from Line B to the leading part of the fetal head, and its measured value corresponds to the TUS station (Fig. 2). TUS was performed during labour using a Voluson P8 ultrasound machine (GE Healthcare, Milwaukee, Wisconsin, USA).

### Statistical analyses

The following data were included in the analyses: maternal baseline characteristics, including gravidity, age, height, body mass index at delivery, and GA at delivery; intrapartum TUS findings before forceps delivery during the intermittent phase of labour and at the time of labour; and delivery outcomes, including the duration of the first and second stages of labour, amount of blood loss, birth weight, Apgar score, and umbilical arterial blood gas pH.

Receiver operating characteristic (ROC) curve analysis was used to evaluate the performance of the classification model at all classification thresholds. The sensitivity and specificity of the TUS station for difficult forceps delivery were calculated, and the maximum values for the area under the ROC curve (AUC) were determined. The AUC values provided an aggregate measure of performance across all possible classification thresholds.

Statistical analyses were performed using JMP 16 software (SAS Institute Inc., Cary, North, USA). Continuous variables were reported as means ± standard deviations and compared using the Student’s t-test. Categorical variables were reported as percentages and were compared using Fisher’s exact test. Statistical significance was set at p < 0.05.

## Results

### Comparisons of the TUS station with DVE findings and the AoP

**Fig. 3:**
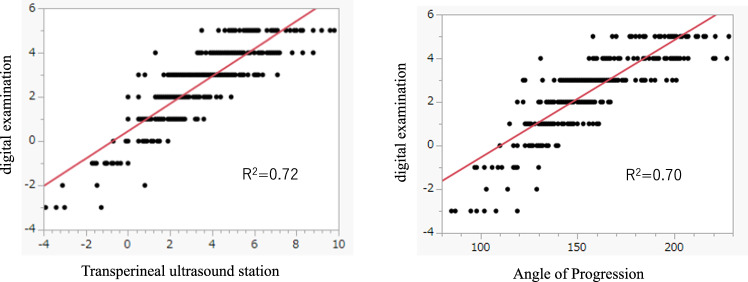
Relation between digital examination and transperineal ultrasound station or angle of progression. Plot of the distribution and relationship between the DVE findings and TUS station or the AoP. The AoP and TUS station correlated with DVE findings to the same extent

The participants were assessed for a descending fetal head in the range of station –3 to + 5 on DVE. The correlation between the TUS station and the DVE findings, and that between the AoP and the DVE findings, are shown in Fig. 3. The obstetricians, who were blinded to all case results, obtained and recorded the DVE findings, TUS station, and AoP, followed by between-parameter comparisons.

Figure 3 shows the distribution and relationship between the TUS station and DVE findings and the relationship between the AoP and DVE findings in images during the second stage of labour. The AoP (R^2^ = 0.70) and TUS stations (R^2^ = 0.72) both correlated with DVE findings to the same extent.

### Prediction of the difficulty of forceps delivery using the TUS station

**Fig. 4:**
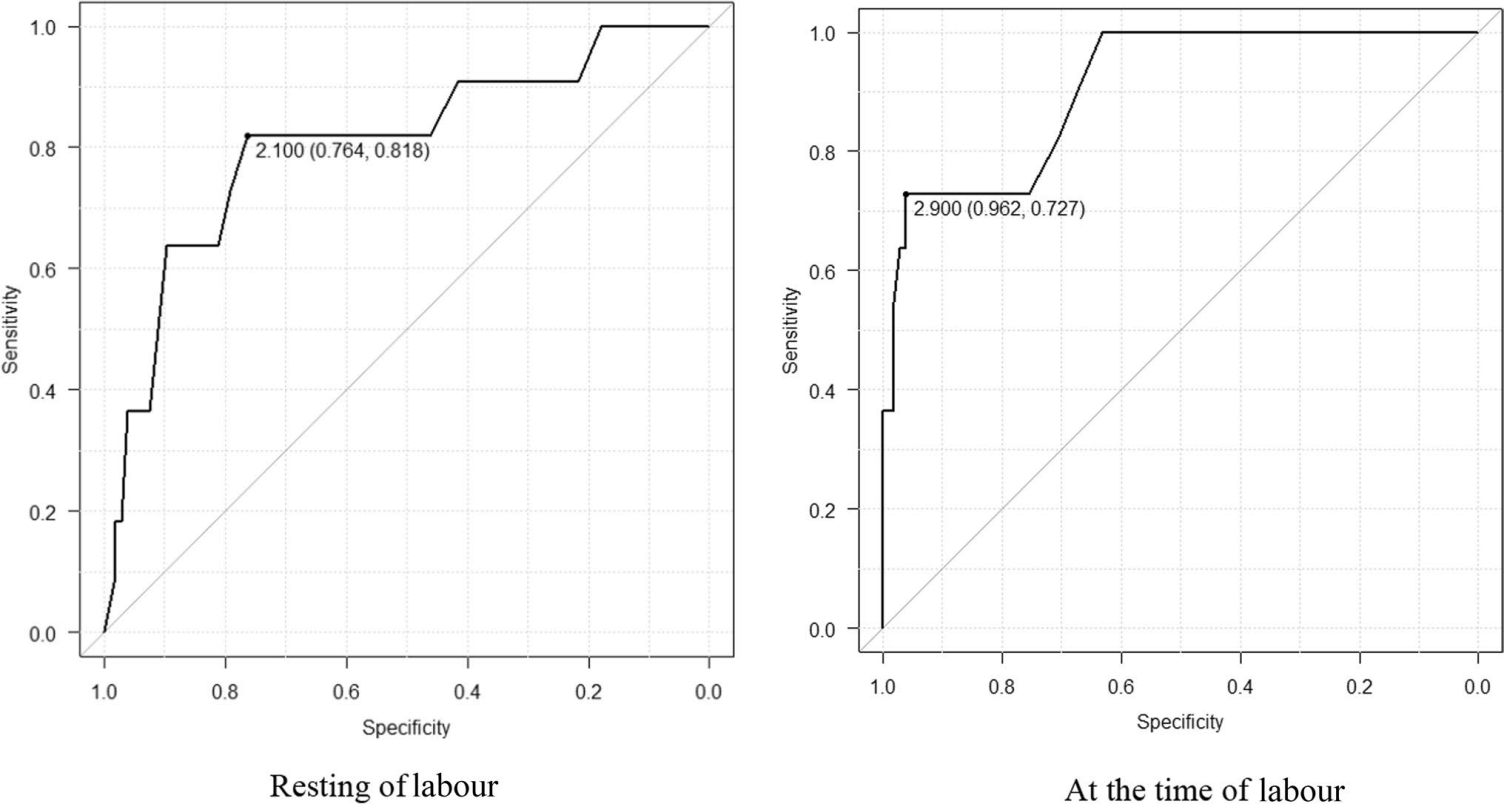
Performance of transperineal ultrasound station for successful forceps delivery. Receiver operating characteristic (ROC) curve for intrapartum transperineal ultrasonographic (TUS) findings in the resting phase and at the time of labour. The TUS station cut-off value is + 2, with the maximum area under the ROC curve (AUC) being 0.9 (95% confidence interval [CI]: 0.73–0.96) at the resting phase of labour. The TUS cut-off value was + 2.9, with the maximum value of the area under the ROC curve (AUC) being 0.8 (95% CI 0.65–0.96) at the time of labour

**Fig. 5:**
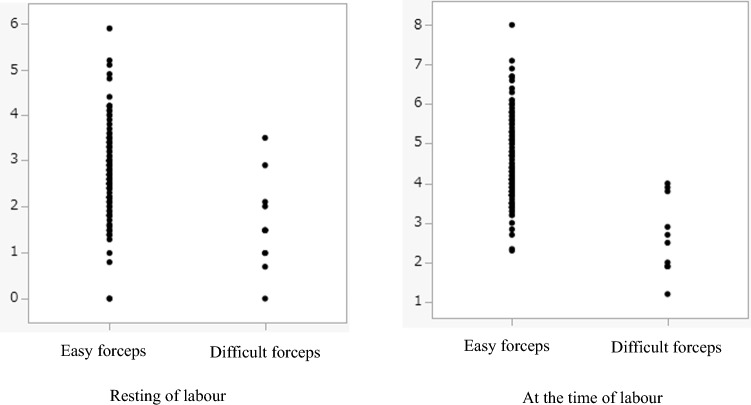
Threshold of TUS station for successful forceps delivery. The TUS stations at attempted forceps deliveries were plotted. The TUS stations of successful forceps deliveries were + 3.5 at the resting phase and + 4.0 at the time of labour

During the study period, 117 forceps deliveries were performed (11 difficult and 106 easy forceps deliveries). All specialist obstetricians who performed forceps deliveries were members of The Japanese Society of Obstetrics and Gynecology and the Japanese Society of Perinatal and Neonatal Medicine. Maternal characteristics, delivery outcomes, and intrapartum TUS findings before forceps delivery are shown in Tables 1, 2, and 3. Table 3 shows a significant difference in the intrapartum TUS findings and AoP of difficult forceps deliveries compared to those of the easy forceps deliveries. The TUS station at any time of labour (resting and labour) was significantly different between the two groups. The fetal head descent distance from TUS station ± 0 was significantly longer in the easy forceps delivery cohort than in the difficult forceps delivery cohort (4.6 ± 0.1 vs. 2.6 ± 0.3, p < 0.001). Figure 4 shows the performance of the TUS station classification model for successful forceps delivery. Specifically, it shows the ROC curve for intrapartum TUS findings in the resting phase of labour and at the time of labour. During the resting phase of labour, the TUS cut-off value for successful forceps delivery was TUS station + 2, with a sensitivity of 85%; the AUC value was 0.9 (95% confidence interval [CI]: 0.73–0.96). At the time of labour, the TUS cut-off value for successful forceps delivery was TUS station + 2.9, with a sensitivity of 85%; the AUC value was 0.8 (95% CI 0.65–0.96). The thresholds of the TUS station for definitely successful forceps delivery were + 3.5 at the resting phase and + 4.0 at the time of labour (Fig. 5).

**Table 1:** Maternal background

Variable	Difficult forceps, n = 11	Easy forceps, n = 106	P-value
Maternal age (years)	34.4 ± 1.4	35.2 ± 0.5	0.60
Height (cm)	160.5 ± 0.5	159.2 ± 1.6	0.44
Weight at delivery (kg)	63.2 ± 2.4	62.0 ± 0.7	0.61
Number of deliveries (mean)	0	0 (0–3)	0.19
Gestational age (weeks)	38.9 ± 0.7	39.0 ± 1.2	0.74

**Table 2:** Delivery outcomes

Variable	Difficult forceps (n = 11)	Easy forceps (n = 106)	P-value
Duration of first stage (min)	399.0 ± 350.9	575.6 ± 42.2	0.20
Duration of second stage (min)	130.2 ± 96.1	163.5 ± 164.7	0.22
Amount of blood loss (ml)	1351.1 ± 197.8	805.4 ± 63.7	0.009
Birth weight (g)	3303.0 ± 109.5	3147.5 ± 35.2	0.91
Umbilical artery blood gas (pH)	7.29 ± 0.01	7.30 ± 0.004	0.22
Apgar score 1 min/5 min	8 (6–9)/9 (7–10)	8 (4–9)/9 (7–10)	0.15

**Table 3:** Intrapartum perineal ultrasound findings

Variable	Difficult forceps (n = 11)	Easy forceps (n = 106)	P-value
TUS station			
Rest phase	1.6 ± 0.3	2.7 ± 0.1	< 0.001
Labour	2.6 ± 0.3	4.6 ± 0.1	< 0.001
ΔTUS	1.0 ± 0.3	1.8 ± 0.1	0.004
AoP			
Rest phase	124.0 ± 20.0	150.9 ± 13.7	< 0.01
Labour	142.8 ± 18.1	173.8 ± 15.7	< 0.01
ΔAoP	18.8 ± 15.9	22.9 ± 12.9	0.1

*TUS* transperineal ultrasound station, ΔTUS = (TUS at the time of labour)—(TUS at the rest phase of labour)

*AoP* angle of progression, ΔAoP = (AoP at the time of labour)—(AoP at the rest phase of labour)

## Discussion

In the present study, we investigated the relationships among the TUS station, DVE findings, and AoP with respect to the degree of descent of the fetal head. The TUS station and AoP showed similar correlations with the DVE findings because both methods quantify the degree of fetal head descent. In the quantitative assessment of fetal head descent, the TUS station showed a correlation with DVE findings, similar to its correlation with AoP. Although the AoP indicates the descent of the fetal head in terms of angle and was converted to the station, our TUS station indicates the descent in terms of distance from the descent of the fetal head, which serves as a more intuitive indicator, providing a clear and understandable representation of the actual DVE.

The AoP has the advantage of being a simple procedure, whereas TUS station needs one more step than the AoP procedure to measure the fetal head station. The arrow C of the TUS measurement procedure represents HD; thus, the TUS station gives us not only the fetal head station but also the head direction, which is important for assessing the difficulty of vaginal birth [12]. While HD is intended to indicate the direction of head progression, determining the direction of HD might be surprisingly challenging for those who are less experienced with transperineal ultrasound. However, since TUS station requires drawing a line during measurement, it might make understanding HD easier for such users [12, 13]. In the future, if the line drawing for the TUS station becomes automated, similar to that for the AoP, it will only be necessary to measure the advanced portion of the fetal head.

Furthermore, regarding the CT analyses of non-pregnant women, Tutschek et al. defined station ± 0 as the parallel plane between the vertical plane of the pubic symphysis and the plane of Hodge, which passes through the ischial spines [14, 15]. Armbrust et al. similarly utilized pelvic CT images from 23 non-pregnant women to evaluate the distance of the interspinous plane as a parallel line to the infrapubic line in two-dimensional intrapartum translabial ultrasound (2D ITU) using 3D CT and digital reconstruction [11]. They reported that the mean distance between the infrapubic plane and the tip of the ischiadic spine was 32.35 (± 4.46) mm (range, 23–38 mm). The average distance between the infrapubic plane and the tip of the ischial spine was reported to have a significant correlation with height but no significant correlation with weight. Meanwhile, we defined station ± 0 from radiographic pelvic measurements using the Guthmann method in 501 Japanese patients with singleton cephalic pregnancies during the third trimester. There is no correlation with maternal height or weight because the TUS station is defined by an angle. Although Tutschek et al. suggest that the curved birth canal should be considered for clinical ITU evaluation, our TUS station can be used to evaluate curved birth canal [11].

In the previous study by Tutschek et al., CT images of non-pregnant females were used [11]. Pregnancy is known to cause pelvic changes. The abdomen is vastly enlarged by fetal growth, and the resulting postural changes, particularly the pelvic alignment changes associated with the augmentation of lumbar kyphosis, place a load on the sacroiliac joint [16, 17]. Accordingly, it is important to consider and adjust for differences in the pelvic shape between pregnant women in the third trimester and non-pregnant women. Moreover, it is important to consider ethnic differences because the pelvic morphology of women of European descent differs from that of women of Japanese descent with a flat pelvic morphology [18].

Various methods have been used to evaluate and report station ± 0. However, the ISUOG Guidelines [7] emphasize that digital examination cannot be replaced with intrapartum ultrasound; instead, they advocate for their complementary use, where intrapartum ultrasound is recommended as supplementary, not a substitute for clinical examination [10]. The TUS station in the difficult forceps delivery cohort was significantly higher than that in the easy forceps delivery cohort at the rest phase of labour and at the time of labour. Based on the ROC curve shown in Fig. 4, the TUS cut-off values were useful in determining whether forceps delivery could be performed safely, and these cut-off values were considered clinically feasible. DVE is sometimes subjective and lacks objectivity [3–6]. Fetal distress can suddenly occur, and it may be necessary to decide on instrumental delivery in isolation. In such situations, TUS may support decision-making for forceps delivery with only DVE.

This study had some limitations. First, the labour-management providers varied, and different obstetricians and midwives applied different management approaches, which may have influenced the delivery outcomes. Second, this was a single-center study. As labour management differs in other obstetric facilities, our findings may not be applicable to these facilities. Finally, this study was conducted in a Japanese population; therefore, studies in other ethnic populations are warranted to validate our findings.

## Conclusions

We investigated the correlation between the TUS station and DVE findings or the AoP and investigated its predictive utility for the difficulty of forceps delivery. In the quantitative assessment of fetal head descent, the TUS station showed almost the same threshold as that of DVE findings. Therefore, it was found to be a useful parameter for determining labour progression and confirming the station determined based on DVE. Furthermore, to the best of our knowledge, this is the first study to demonstrate the predictive utility of the TUS station for the difficulty of forceps delivery. Our findings may contribute to the decision-making of obstetricians regarding whether to perform forceps delivery, thus preventing maternal and fetal complications. A future study is planned to investigate whether TUS station in deliveries with rotational abnormalities may allow objective evaluation of the extraction of difficult vaginal delivery cases.

## Data Availability

The data that support the findings of this study are available from the corresponding author upon reasonable request.
